# Long-term survival after ALK-TKI resistance through immunotherapy combined with chemotherapy in ALK-positive non-small cell lung cancer: a case report

**DOI:** 10.3389/fonc.2025.1732738

**Published:** 2025-12-12

**Authors:** Caihong Fu, Lei Yang, Hui Qiao, Xiting Liu, Jiexin Wang

**Affiliations:** 1 Department of Respiratory Oncology, Gansu Provincial Cancer Hospital, Lanzhou, China; 2 Department of Medical Oncology, First Hospital of Lanzhou University, Lanzhou, China; 3 Department of Pharmacy, Gansu Provincial Cancer Hospital, Lanzhou, China

**Keywords:** ALK fusion positivity, co-mutation, immunotherapy, target therapy, tislelizumab, TP53

## Abstract

Anaplastic lymphoma kinase (ALK) gene fusions represent one of the most common driver mutations in non-small cell lung cancer (NSCLC). Currently, targeted drug options for ALK fusion-positive patients exhibit diversity, with third-generation drugs achieving a 5-year progression-free survival (PFS) rate exceeding 60%. Nevertheless, for advanced patients, targeted therapy serves as palliative treatment, with the inevitability of disease progression and the development of therapeutic resistance over time. Yet, there are no unified standards regarding progression patterns, resistance mechanisms, or subsequent treatment strategies. The use of immune checkpoint inhibitors (ICIs) in ALK fusion-positive patients remains highly controversial, and clinical data on whether immune checkpoint inhibitor-based therapy can be administered sequentially after progression on ALK Tyrosine Kinase Inhibitors (ALK-TKI) treatment are limited. This case presents a patient with ALK fusion-positive disease who progressed rapidly after receiving first-generation ALK-TKI followed by second-generation ALK-TKI therapy, along with radiotherapy targeting primary and metastatic lesions. Following chemotherapy combined with immunotherapy, the patient achieved a median response duration of more than 45 months.

## 1 Introduction

In 2007, Japanese researchers first reported ALK fusion positivity as an oncogenic driver gene in non-small cell lung cancer (NSCLC) (1). A nationwide multicenter retrospective clinical study found that the incidence of ALK fusion in NSCLC was 6.7% (2). Although the advent of targeted drugs has continuously extended the survival period for these patients, there is currently insufficient evidence to determine the exact efficacy of chemotherapy-based treatments or sequential other ALK-TKI therapies for patients resistant to second-generation ALK-TKI. The clinical benefit of immune checkpoint inhibitors (ICIs), which have been extensively studied, remains uncertain for ALK fusion-positive patients. In previous studies, immunotherapy combined with bevacizumab and chemotherapy demonstrated benefits in PFS and overall survival (OS) for ALK fusion-positive NSCLC, but the enrolled sample size was small (3). Real-world and retrospective clinical studies confirm that immune monotherapy offers very limited clinical benefit in ALK fusion-positive patients (4, 5). Previous studies have confirmed that programmed death-ligand 1 (PD-L1) expression can serve as one of the predictive factors for the efficacy of immunotherapy (6). Relevant studies indicate that PD-L1 is highly expressed in ALK fusion-positive lung cancer (7, 8). However, whether PD-L1 expression can serve as a predictive factor for immunotherapy efficacy in ALK fusion-positive patients remains highly controversial, and no large-scale clinical studies have confirmed this. This case presents a patient with ALK fusion-positive status, concurrent TP53 mutation, and high PD-L1 expression. After receiving first- and second-line ALK-TKI targeted therapy combined with local radiotherapy, the patient experienced a short disease remission period. After progression, third-line chemotherapy combined with immunotherapy ultimately resulted in sustained long-term disease remission.

## 2 Case presentation

**Figure 1:**
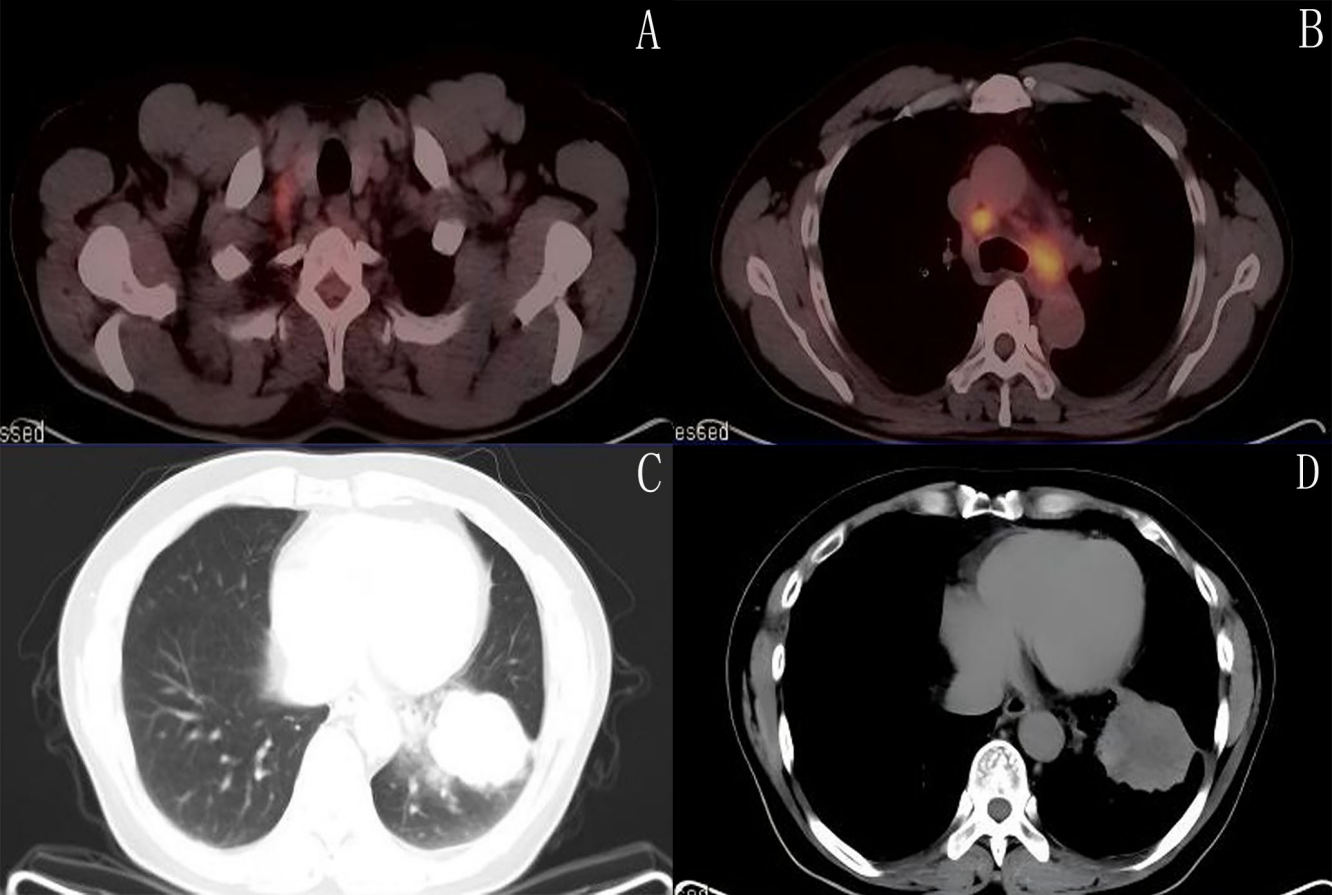
Baseline Image (PET-CT on March 25, 2021). **(A)** Right supraclavicular lymph node metastasis. **(B)** Mediastinal and left hilar lymph node metastasis. **(C)** Pulmonary window:solid mass in the left lower lobe of the lung. **(D)** Diaphragmatic window: solid mass in the left lower lobe of the lung.

The patient is a 44-year-old male who presented with a cough and blood-streaked sputum lasting one month in February 2021. History of smoking for 20 years, approximately 30 cigarettes daily. Chest CT at another hospital on March 4th revealed a solid mass in the left lower lobe of the lung. Electronic bronchoscopy revealed occupation at the anterior basal segment of the left lower lobe inner base segment, with pathology suggesting adenocarcinoma. Immunohistochemistry results: ALK (D5F3 Ventana)(+), AE1/AE3 (+), CK7(+), CK5/6 (–), NapsinA (partial+), P40 (–), P63 (–), TTF-1(+), Ki-67 local index approximately 50%. On March 25, 2021, PET-CT findings: 1. Solid mass in the left lower lobe of the lung (4.1cm × 3.0cm × 5.1cm) (**Figures 1C, D**), consistent with lung cancer and obstructive atelectasis based on pathology. 2. Multiple enlarged lymph nodes in the right supraclavicular region (**Figure 1A**), mediastinum (zones 2L, 4R, 5, 6, 7) (**Figure 1B**), and left hilar region(**Figure 1B**), suggestive of metastatic lesions. The preliminary diagnosis: Adenocarcinoma of the left lower lobe of the lung, cT3N3M0 Stage IIIC (AJCC 8th edition staging), ECOG score of 1.

The patient underwent three-dimensional conformal radiation therapy for the primary lung lesion (DT:46Gy/23f/5w) from April 15 to May 19, 2021, at an external hospital. During this period, Second-generation sequencing genetic test results reported ALK fusion positivity combined with TP53 (exon5, c.473G>A,p R158H) mutation,PD-L1 (22C3) TPS approaching 85%. The patient began receiving molecular targeted therapy on April 29, 2021, with a prescription of crizotinib 250mg twice daily. According to the Response Evaluation Criteria in Solid Tumors (RECIST) version 1.1, the tumor evaluation showed reduced stable disease (SD), and the patient experienced a decrease in appetite during medication use. August 2021 evaluation showed disease progression (PD), with enlargement of pulmonary lesions and a new metastatic tumor in the left adrenal gland, complicated by intra-tumoral hemorrhage. The treatment switched to alectinib hydrochloride 600mg twice daily from August 7, 2021, and involved radiotherapy for the left adrenal gland lesion (DT: 40Gy/20f/5w) from August 6 to September 14, 2021. The best response achieved was SD. Second-line treatment duration is nearly 5.5 months.

**Figure 2:**
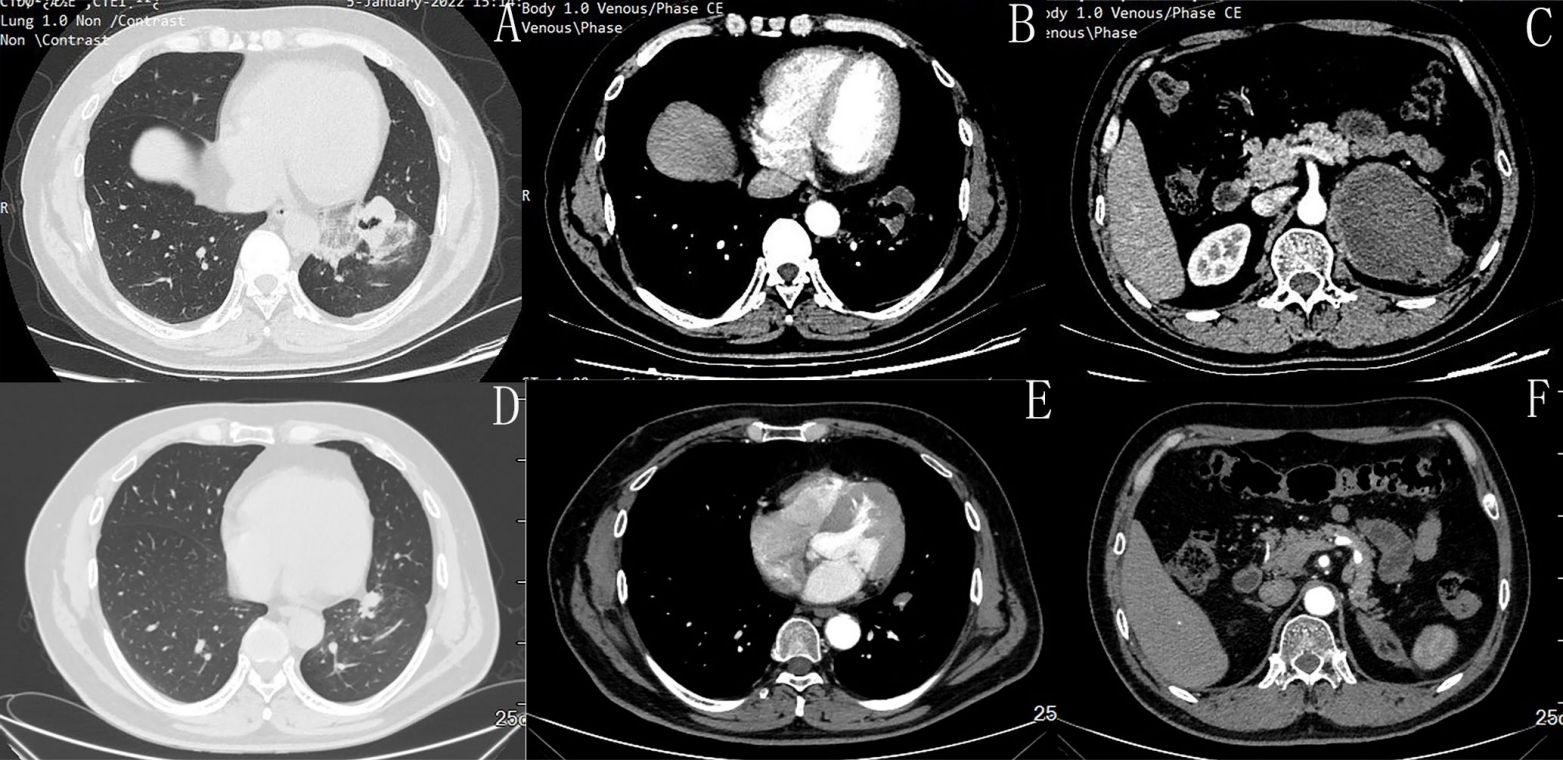
Images before chemotherapy combined with immunotherapy(January 2022). **(A)** Pulmonary window:primary tumor in the lower lobe of the left lung, measuring 4.9 cm × 3.1 cm. **(B)** Pulmonary window: left lower lobe lesion. **(C)** Adrenal metastatic lesion, measuring 9.8 cm × 7.2 cm. **(D–F)** The most recent CT scan images (September 9, 2025). primary tumor in the lower lobe of the left lung and adrenal metastatic lesion.

**Figure 3:**
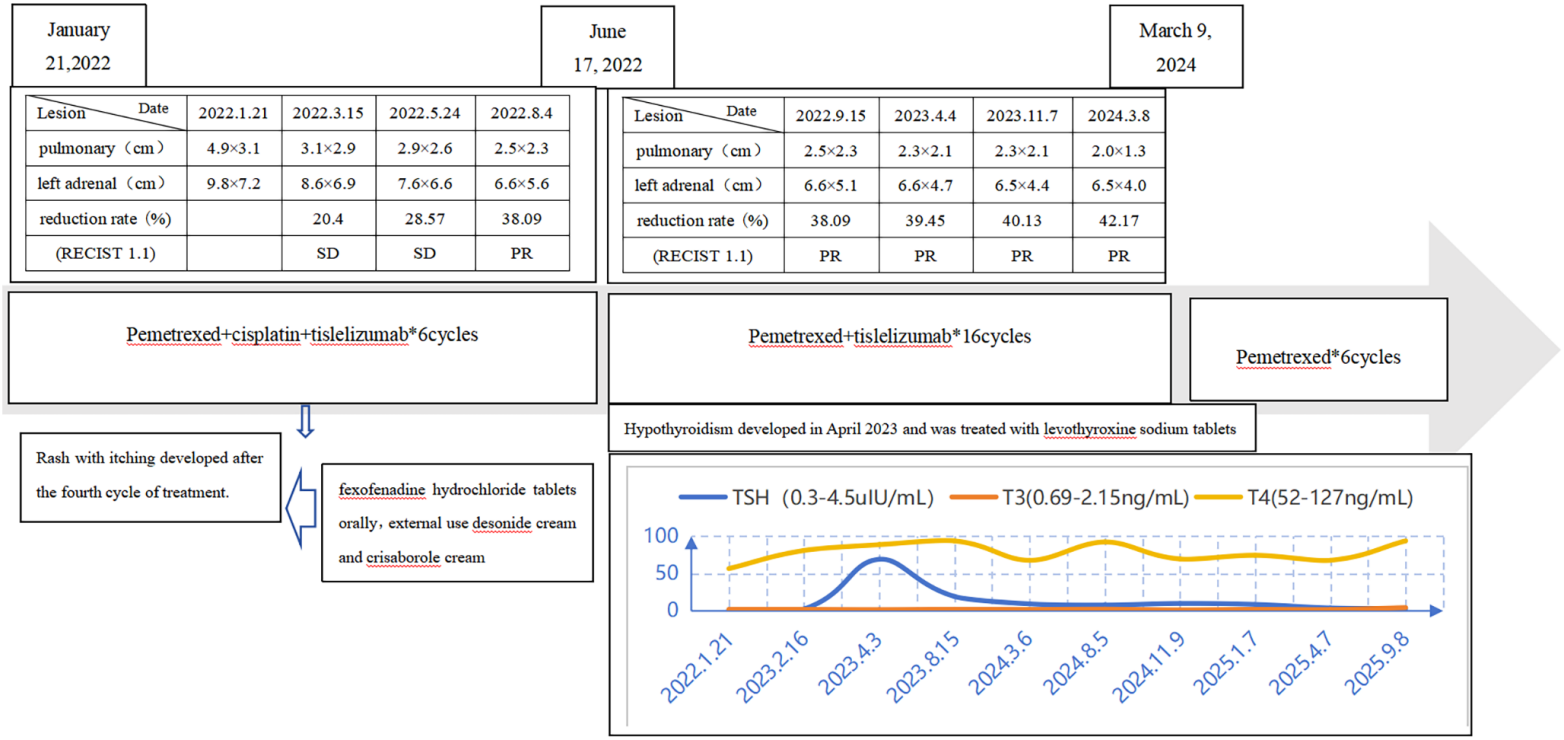
Flowchart for third-line immunotherapy combined with chemotherapy and subsequent maintenance therapy.

**Figure 4:**
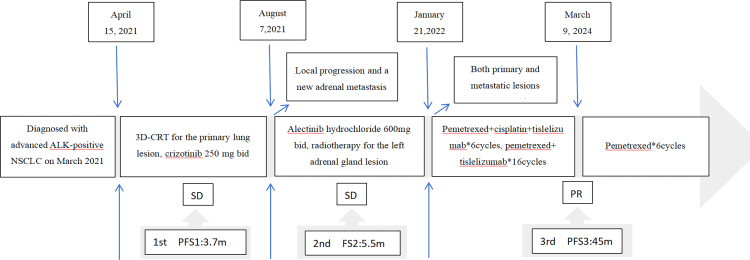
Patient treatment course.

In January 2022, imaging evaluation of disease progression, with an increase in the size of the pulmonary lesion (from 3.1cm × 2.1cm to 4.9cm × 3.1cm) and the left adrenal metastasis (from 5.7cm × 6.0cm to 9.8cm ×7.2cm) (**Figures 2A–C**). Initial presentation at our hospital. Following multidisciplinary team discussion, the patient received 6 cycles of Pemetrexed (500mg/m^2^, Day 1) combined with cisplatin (75mg/m^2^, Day 1) plus tislelizumab (200mg, Day 1) therapy from January 21, 2022, to June 17, 2022 (Cycles 1-6). Subsequently, maintenance therapy with pemetrexed (500mg/m^2^, Day 1) plus tislelizumab (200mg, Day 1) was administered from August 10, 2022, to March 9, 2024 (16 cycles), during which response was assessed as PR according to RECIST 1.1 criteria. During treatment, Grade II hypothyroidism and Grade II rash developed. For the rash, administer fexofenadine hydrochloride tablets orally and external use desonide cream and crisaborole cream topically, once symptoms have subsided, and most of the rash has faded, with no impact on subsequent medication use. Against hypothyroidism, the final adjustment of levothyroxine sodium tablets dosage to 100ug daily resulted in a TSH level below 10 uIU/mL, both improving with symptomatic management. A PET-CT scan performed at another hospital on December 21, 2023, showed: After treatment of malignant tumors in the left lung, a nodular, slightly hyperdense lesion with an oblique fissure in the anterior basal segment of the left lower lobe is noted beneath the pleura, and no obvious glucose uptake was observed. A patchy soft tissue density shadow is observed in the left adrenal region, also showing no significant glucose uptake. No significantly enlarged or hypermetabolic lymph nodes are identified in the mediastinum and bilateral hilar regions. Collectively, these findings indicate that the tumor has remained in sustained remission following treatment. Since May 15, 2024, the patient has received 6 cycles of pemetrexed (500mg/m^2^, Day 1) monotherapy maintenance treatment (**Figure 3**). Imaging studies continue to demonstrate remission status (**Figures 2D, E, F**), and the patient’s ECOG performance status remains at 0. The timeline of the treatment process is shown in **Figure 4**.

## 3 Discussion

At the initial diagnosis, stage III NSCLC represents a highly heterogeneous group of diseases. In this case, PET-CT findings revealed multiple lymph node metastases in the supraclavicular and mediastinal regions, with preliminary staging indicating unresectable stage IIIC disease. According to NCCN and CSCO guidelines, the standard regimen involves immunotherapy consolidation treatment following concurrent chemoradiation therapy, based on the results of the PACIFIC study (9). The recommended radiotherapy dose is 60–66 Gy. The patient received radiotherapy as the primary treatment without prior systemic therapy at initial diagnosis, and the radiation dose administered did not reach curative levels. During radiotherapy, genetic testing results indicated ALK fusion positivity combined with a TP53 mutation. Crizotinib targeted therapy was administered promptly during radiotherapy. However, the clinical benefit from first-line treatment was very limited, and distant metastases developed within a short period. Second-line therapy switched to second-generation ALK-TKI alectinib hydrochloride molecular targeted therapy, combined with local radiotherapy for adrenal metastases. However, the patient’s disease response duration remained only 5.5 months. Potentially related to the presence of TP53 mutations. Patients with ALK fusions and concurrent TP53 mutations exhibit reduced responsiveness to ALK-TKI targeted therapy. Previous studies have confirmed that patients with ALK fusions and concurrent TP53 mutations exhibit poorer PFS, OS, and overall response rates (ORR) compared to those with wild-type TP53 (10, 11). A study from Germany, which included 216 patients with ALK-positive stage IIIB/IV NSCLC, found that the incidence of TP53 co-mutation with ALK fusion was 23.8%. In patients with co-mutations, both median PFS (3.9 vs 10.3 months) and median OS (15.0 vs 50.0 months) were significantly shorter compared to those without co-mutations. Whether receiving chemotherapy, targeted therapy, or chemotherapy combined with targeted therapy, the prognosis for those with TP53 co-mutations was significantly worse (12). Another real-world dataset from the US GuardantINFORM database suggests that among ALK fusion-positive NSCLC patients receiving second- or third-generation ALK-TLI therapy, those with concurrent TP53 mutations experienced significantly shorter Time to Disease Progression (TTD) compared to those without detectable TP53 mutations (13.1 months vs. 27.6 months, HR = 1.53, 95% CI: 1.07-2.19, p=0.0202) (13). However, the association between TP53 mutations and the efficacy of immune checkpoint inhibitors remains unclear.

There are currently no definitive biomarkers available to predict the efficacy of immunotherapy. Previous studies have demonstrated that high tumor mutational burden (TMB) and high PD-L1 expression may be positively correlated with treatment efficacy (14, 15). Nevertheless, relevant studies have primarily included patients with negative driver gene status. For those with positive driver genes, especially those harboring fusion mutations, the efficacy of immune checkpoint inhibitors is even inferior to systemic chemotherapy (16). In a multicenter retrospective clinical study, 23 patients with ALK fusion-positive NSCLC received ICIs monotherapy. The ORR was 0%, median PFS was 2.5 months (95% CI: 1.5-3.7), and median OS was 17.0 months (95% CI: 3.6-Not Reached) (4). Another real-world retrospective study enrolled 83 ALK fusion-positive NSCLC patients, of whom 74 received monotherapy with an immune checkpoint inhibitor. The PFS for patients receiving an immune checkpoint inhibitor followed by ALK-TKI was 3.9 months, whereas PFS for those receiving ALK-TKI followed by an immune checkpoint inhibitor was only 1.5 months (5). Therefore, it remains uncertain whether ALK fusion-positive patients can benefit from immunotherapy after ALK-TKI treatment. This patient presented with ALK fusion positivity, concurrent TP53 mutation, and high PD-L1 expression. The patient derived very limited benefit from ALK-TKI therapy. Switching to chemotherapy combined with immunotherapy resulted in disease remission with prolonged duration. This suggests that sequential immunotherapy following targeted therapy may confer survival benefit for patients with ALK fusion positivity and high PD-L1 expression. Unfortunately, the patient did not undergo TMB testing, making it impossible to more accurately predict the efficacy of immunotherapy. Consequently, it is not possible to confirm the correlation between this patient’s clinical benefit from immunotherapy and TMB status. Should disease progression occur subsequently, it will be necessary to retest the ALK fusion mutation status, PD-L1 expression level, and TMB status. However, prospective clinical studies are needed to further validate this approach.

In NSCLC cell lines, the EML4-ALK fusion protein induces PD-L1 expression. A Ba/F3 cell model was constructed by transfecting an EML4-ALK expression vector. Immunoblotting analysis using antibodies against phosphorylated ALK and total ALK confirmed the expression and phosphorylation of EML4-ALK in transfected cells. Quantitative RT-PCR analysis demonstrated that EML4-ALK expression significantly increased PD-L1 mRNA abundance in Ba/F3 cells. Flow cytometry analysis revealed that EML4-ALK also elevated PD-L1 expression levels on the surface of Ba/F3 cells, while alectinib markedly inhibited this effect. These findings indicate that the EML4-ALK fusion protein positively regulates PD-L1 expression at both the mRNA and protein levels (17). Moreover, PD-L1 expression is regulated by both the PI3K-AKT and MEK-ERK signaling pathways, and EML4-ALK can also upregulate PD-L1 expression through STAT3 and HIF-1α (17, 18). Similarly, in surgically resected non-small cell lung cancer specimens, the EML4-ALK fusion protein was observed to upregulate PD-L1 expression (17, 19). Radiation therapy may induce DNA damage and cellular stress, activating multiple signaling pathways, including EGFR and JAK/STAT. The activation of these pathways rapidly leads to the upregulation of PD-L1 at both the mRNA and protein levels. Established research confirms that in radiotherapy combined with immunotherapy, radiotherapy activates the immune system and upregulates PD-L1, while ICIs counteract this adaptive resistance, unleashing T-cell function and including the abscopal effect (20, 21). ALK-TKIs reduce PD-L1 expression levels by inhibiting ALK kinase activity and blocking its downstream PI3K-AKT and MEK-ERK signaling pathways (17). TP53 mutations drive high PD-L1 expression on tumor cells through multiple pathways, including the release of miR-34a, genomic instability, and increased TMB. This makes such patients more likely to benefit from PD-1/PD-L1 inhibitor therapy (22–24).

This patient harbors a TP53 mutation, and following radiotherapy for lung and adrenal metastases, PD-L1 overexpression may occur. However, prior treatment with first- and second-generation ALK-TKIs may reduce PD-L1 expression. Theoretically, the concurrent administration of targeted therapy and local radiotherapy may result in an offsetting effect, with the outcome determined by which effect predominates. This may be influenced by factors such as radiotherapy dose, the type and concentration of ALK inhibitors, and the biological characteristics of tumor cells. Such complex interactions necessitate validation through meticulously designed clinical trials to establish the optimal combination timing, dosage, and sequence. We recommend that this patient undergo a repeat biopsy prior to initiating chemotherapy combined with immunotherapy to assess ALK fusion status, PD-L1 expression, and TMB levels. Additionally, dynamic monitoring of immunological markers should be conducted throughout treatment. Due to financial constraints and the urgent need for clinical symptom improvement, this patient did not undergo a repeat biopsy nor achieve immunologic or genomic testing data during subsequent treatment. Consequently, potential biomarkers that may impact treatment efficacy remain unclear, highlighting heightened demands for future clinical diagnosis and management.

But the predictive value of PD-L1 expression for immunotherapy in ALK-rearranged NSCLC remains unclear. This patient presented with ALK fusion positivity and high PD-L1 expression. During treatment with oral ALK-TKI, disease response duration was brief. After progression on second-generation ALK-TKI, third-generation drugs were unavailable at the time. According to the guidelines, platinum-based chemotherapy may be considered with or without bevacizumab. A real-world retrospective study of ALK-rearranged NSCLC patients receiving ICIs demonstrated a median PFS of 3.9 months in patients who received ICIs prior to ALK-TKI treatment, compared with a median PFS of 1.5 months in patients who received ICIs after ALK-TKI treatment (5). In IMPOWER 150, the four-drug combination ABCP (atezolizumab + bevacizumab + carboplatin + paclitaxel) demonstrated benefits in PFS and OS compared to the BCP regimen in EGFR/ALK-positive cases (3). There is currently a lack of clinical studies on the use of tislelizumab for treating ALK fusion-positive NSCLC. Considering that initial genetic testing indicated high PD-L1 expression, they represent a potential candidate for immunotherapy, and the affordability of medications. Third-line therapy involved tislelizumab combined with chemotherapy, resulting in sustained disease response. A PET-CT scan performed nearly two years after treatment initiation showed no significant glucose uptake in the primary lesion in the left lung or the metastatic lesion in the left adrenal gland. Considering the substantial tumor response post-treatment and the fact that the patient has been off immune checkpoint inhibitors for over a year, the patient remains in sustained remission. This case provides preliminary evidence for the use of immune checkpoint inhibitors in ALK-fusion-positive patients with high PD-L1 expression who progress after TKI therapy. However, prospective clinical studies are still needed to further validate this approach.

## 4 Conclusion

We report a fully documented case of a patient with ALK fusion-positive NSCLC, concurrently harboring a TP53 co-mutation and PD-L1 high expression. Following a brief period of disease control with ALK-TKI combined with local radiotherapy, the patient achieved a disease control duration exceeding 45 months through immune checkpoint inhibitors combined with chemotherapy. This case provides a reference for the use of immune checkpoint inhibitors in patients with ALK fusion-positive NSCLC with concurrent TP53 co-mutations and high PD-L1 expression who progressed after ALK-TKI. However, further prospective clinical studies are needed to validate these findings.

## Data Availability

The raw data supporting the conclusions of this article will be made available by the authors, without undue reservation.

## References

[1] Soda M Choi YL Enomoto M Takada S Yamashita Y Ishikawa S . Identification of the transforming EML4-ALK fusion gene in non-small-cell lung cancer. Nature. (2007) 448:561–6. doi: 10.1038/nature05945, PMID: 17625570

[2] Li L Li W Wu C Xi Y Guo L Ji Y . Real-world data on ALK rearrangement test in Chinese advanced non-small cell lung cancer (RATICAL): a nationwide multicenter retrospective study. Cancer Commun Lond Engl. (2024) 44:992–1004. doi: 10.1002/cac2.12593, PMID: 39016057 PMC11492361

[3] Socinski MA Jotte RM Cappuzzo F Orlandi F Stroyakovskiy D Nogami N . Atezolizumab for first-line treatment of metastatic nonsquamous NSCLC. N Engl J Med. (2018) 378:2288–301. doi: 10.1056/NEJMoa1716948, PMID: 29863955

[4] Mazieres J Drilon A Lusque A Mhanna L Cortot AB Mezquita L . Immune checkpoint inhibitors for patients with advanced lung cancer and oncogenic driver alterations: results from the IMMUNOTARGET registry. Ann Oncol Off J Eur Soc Med Oncol. (2019) 30:1321–8. doi: 10.1093/annonc/mdz167, PMID: 31125062 PMC7389252

[5] Jahanzeb M Lin HM Pan X Yin Y Baumann P Langer CJ . Immunotherapy treatment patterns and outcomes among ALK-positive patients with non-small-cell lung cancer. Clin Lung Cancer. (2021) 22:49–57. doi: 10.1016/j.cllc.2020.08.003, PMID: 33250347

[6] Reck M Rodríguez-Abreu D Robinson AG Hui R Csőszi T Fülöp A . Pembrolizumab versus chemotherapy for PD-L1-positive non-small-cell lung cancer. N Engl J Med. (2016) 375:1823–33. doi: 10.1056/NEJMoa1606774, PMID: 27718847

[7] Schabath MB Dalvi TB Dai HA Crim AL Midha A Shire N . A molecular epidemiological analysis of programmed cell death ligand-1 (PD-L1) protein expression, mutations and survival in non-small cell lung cancer. Cancer Manag Res. (2019) 11:9469–81. doi: 10.2147/CMAR.S218635, PMID: 31819612 PMC6844199

[8] Rangachari D VanderLaan PA Shea M Le X Huberman MS Kobayashi SS . Correlation between classic driver oncogene mutations in EGFR, ALK, or ROS1 and 22C3-PD-L1 ≥50% Expression in lung adenocarcinoma. J Thorac Oncol Off Publ Int Assoc Study Lung Cancer. (2017) 12:878–83. doi: 10.1016/j.jtho.2016.12.026, PMID: 28104537 PMC5403565

[9] Spigel DR Faivre-Finn C Gray JE Vicente D Planchard D Paz-Ares L . Five-year survival outcomes from the PACIFIC trial: durvalumab after chemoradiotherapy in stage III non-small-cell lung cancer. J Clin Oncol Off J Am Soc Clin Oncol. (2022) 40:1301–11. doi: 10.1200/JCO.21.01308, PMID: 35108059 PMC9015199

[10] Lara-Mejía L Cardona AF Mas L Martin C Samtani S Corrales L . Impact of concurrent genomic alterations on clinical outcomes in patients with ALK-rearranged NSCLC. J Thorac Oncol Off Publ Int Assoc Study Lung Cancer. (2024) 19:119–29. doi: 10.1016/j.jtho.2023.08.007, PMID: 37572870

[11] Qin K Hou H Liang Y Zhang X . Prognostic value of TP53 concurrent mutations for EGFR- TKIs and ALK-TKIs based targeted therapy in advanced non-small cell lung cancer: a meta-analysis. BMC Cancer. (2020) 20:328. doi: 10.1186/s12885-020-06805-5, PMID: 32299384 PMC7164297

[12] Kron A Alidousty C Scheffler M Merkelbach-Bruse S Seidel D Riedel R . Impact of TP53 mutation status on systemic treatment outcome in ALK-rearranged non-small-cell lung cancer. Ann Oncol Off J Eur Soc Med Oncol. (2018) 29:2068–75. doi: 10.1093/annonc/mdy333, PMID: 30165392 PMC6225899

[13] Parikh K Dimou A Leventakos K Mansfield AS Shanshal M Wan Y . Impact of EML4-ALK variants and co-occurring TP53 mutations on duration of first-line ALK tyrosine kinase inhibitor treatment and overall survival in ALK fusion-positive NSCLC: real-world outcomes from the guardantINFORM database. J Thorac Oncol Off Publ Int Assoc Study Lung Cancer. (2024) 19:1539–49. doi: 10.1016/j.jtho.2024.07.009, PMID: 39019326

[14] Reck M Rodríguez-Abreu D Robinson AG Hui R Csőszi T Fülöp A . Five-year outcomes with pembrolizumab versus chemotherapy for metastatic non-small-cell lung cancer with PD-L1 tumor proportion score ≥ 50. J Clin Oncol Off J Am Soc Clin Oncol. (2021) 39:2339–49. doi: 10.1200/JCO.21.00174, PMID: 33872070 PMC8280089

[15] Mok TSK Wu YL Kudaba I Kowalski DM Cho BC Turna HZ . Pembrolizumab versus chemotherapy for previously untreated, PD-L1-expressing, locally advanced or metastatic non-small-cell lung cancer (KEYNOTE-042): a randomised, open-label, controlled, phase 3 trial. Lancet Lond Engl. (2019) 393:1819–30. doi: 10.1016/S0140-6736(18)32409-7, PMID: 30955977

[16] Negrao MV Skoulidis F Montesion M Schulze K Bara I Shen V . Oncogene-specific differences in tumor mutational burden, PD-L1 expression, and outcomes from immunotherapy in non-small cell lung cancer. J Immunother Cancer. (2021) 9:e002891. doi: 10.1136/jitc-2021-002891, PMID: 34376553 PMC8356172

[17] Ota K Azuma K Kawahara A Hattori S Iwama E Tanizaki J . Induction of PD-L1 expression by the EML4-ALK oncoprotein and downstream signaling pathways in non-small cell lung cancer. Clin Cancer Res Off J Am Assoc Cancer Res. (2015) 21:4014–21. doi: 10.1158/1078-0432.CCR-15-0016, PMID: 26019170

[18] Koh J Jang JY Keam B Kim S Kim MY Go H . EML4-ALK enhances programmed cell death-ligand 1 expression in pulmonary adenocarcinoma via hypoxia-inducible factor (HIF)-1α and STAT3. Oncoimmunology. (2015) 5:e1108514. doi: 10.1080/2162402X.2015.1108514, PMID: 27141364 PMC4839370

[19] Hong S Chen N Fang W Zhan J Liu Q Kang S . Upregulation of PD-L1 by EML4-ALK fusion protein mediates the immune escape in ALK positive NSCLC: Implication for optional anti-PD-1/PD-L1 immune therapy for ALK-TKIs sensitive and resistant NSCLC patients. Oncoimmunology. (2015) 5:e1094598. doi: 10.1080/2162402X.2015.1094598, PMID: 27141355 PMC4839382

[20] Wu CT Chen WC Chang YH Lin WY Chen MF . The role of PD-L1 in the radiation response and clinical outcome for bladder cancer. Sci Rep. (2016) 6:19740. doi: 10.1038/srep19740, PMID: 26804478 PMC4726250

[21] Song X Shao Y Jiang T Ding Y Xu B Zheng X . Radiotherapy upregulates programmed death ligand-1 through the pathways downstream of epidermal growth factor receptor in glioma. EBioMedicine. (2018) 28:105–13. doi: 10.1016/j.ebiom.2018.01.027, PMID: 29396299 PMC5835577

[22] Cha YJ Kim HR Lee CY Cho BC Shim HS . Clinicopathological and prognostic significance of programmed cell death ligand-1 expression in lung adenocarcinoma and its relationship with p53 status. Lung Cancer Amst Neth. (2016) 97:73–80. doi: 10.1016/j.lungcan.2016.05.001, PMID: 27237031

[23] Dong ZY Zhong WZ Zhang XC Su J Xie Z Liu SY . Potential predictive value of TP53 and KRAS mutation status for response to PD-1 blockade immunotherapy in lung adenocarcinoma. Clin Cancer Res Off J Am Assoc Cancer Res. (2017) 23:3012–24. doi: 10.1158/1078-0432.CCR-16-2554, PMID: 28039262

[24] Assoun S Theou-Anton N Nguenang M Cazes A Danel C Abbar B . Association of TP53 mutations with response and longer survival under immune checkpoint inhibitors in advanced non-small-cell lung cancer. Lung Cancer Amst Neth. (2019) 132:65–71. doi: 10.1016/j.lungcan.2019.04.005, PMID: 31097096

